# Supplementary material for: Democratizing social media for health research: LLM-powered data analytics platform for NCDs
Source: PLoS One. 2025 Sep 17;20(9):e0332484. doi: 10.1371/journal.pone.0332484 (PMC12443273; doi:10.1371/journal.pone.0332484)
Supplement: Attachment — Submitted filename: Responses to all comments.pdf [file pone.0332484.s001.pdf]

Author response to all reviewers' comments for manuscript [PONE-D-25-21639R1] - [EMID:cbd38050ce523c16]

We sincerely thank all reviewers for providing insightful and constructive comments that have greatly improved this manuscript. We have carefully considered and addressed all the comments raised by the reviewers. The manuscript has been substantially revised to improve the presentation of our study. The point-to-point responses and explanations for all the revisions are listed in the table below for easy reference. Below, each comment is listed in the left column of the table, and our responses are written in the right column of the table.

Order	comments	Author reply
Editor's comments		
1	Providing a Data Availability Statement that clearly outlines how the data supporting your findings can be accessed.	We have provided a statement on the availability and accessibility of the data supporting our work.<br><br>**Revised manuscript (See line 150-151)**<br>"This study extends previous work by introducing the NCDs Listener, a social listening tool. The code, model development data, and evaluation data can be accessed through the project repository [16]."
2	Sharing all relevant software and code used in the study, ideally through a public repository (e.g., GitHub, Zenodo).	We have shared our software and code via a public repository on Hugging Face, as noted in footnote 1 of the abstract first page. Additionally, we have included supporting data for our work, such as data used to evaluate the communication capabilities of the Dashboard and Generative AI, data used to develop the BERT model, and scraped web data.
3	Ensuring that all materials, including datasets and tools, are accessible without restriction, unless ethical or legal limitations apply.	We certify that all materials, including datasets and tools, are accessible without restriction.

<table>
<tr><th>Order</th><th>comments</th><th>Author reply</th></tr>
<tr><td colspan="3" align="center">Reviewer #1</td></tr>
<tr><td>4</td><td>System Architecture Depth: While Figure 1 has been updated, it still lacks
the technical granularity expected for a platform paper. The diagram needs to show:

- Detailed component interactions and data flow
- Database schema and API integration points
- Specific framework interconnections
- Scalability and performance architecture</td><td>In Figure 1, we have enhanced the depth of the illustration by providing detailed explanations for the types of data flowing into and out of each node, further elaborating on the database used for storing vector data, the application of Google APIs, and the specific tools employed in each component.

Origen manuscript
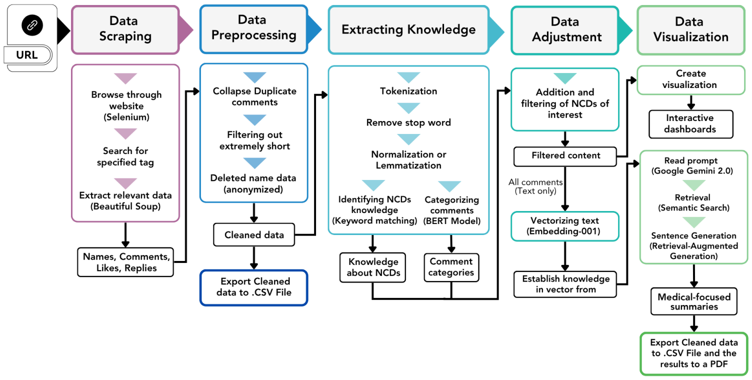

Revised manuscript (Page 6)
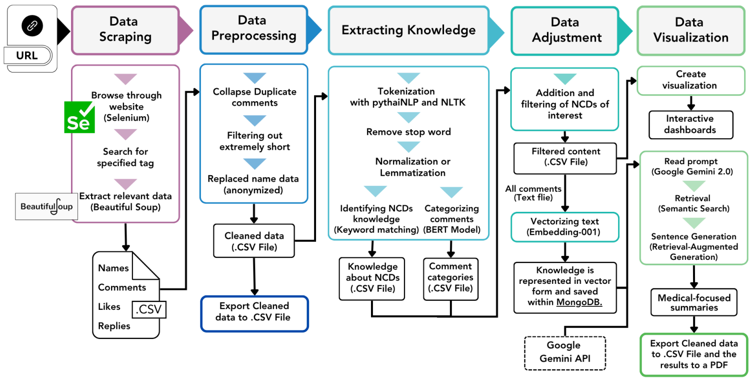
</td></tr>
<tr><td>5</td><td>Evaluation Limitations Persist: Despite statistical justification, the evaluation methodology remains limited:

No comparative</td><td>We have expanded on the performance of our tool and provided a comparative analysis of its functionalities against existing tools in a new section titled 'Performance and Functional Comparison'.

Revised manuscript (line 478-497)
“In this section, we tested our tool with real users, from the data extraction process to image display, using posts containing 30 to 2,000 comments from both Facebook and Reddit. We found that processing on Facebook took</td></tr>
</table>

Order	comments	Author reply
	analysis with existing tools (even basic functionality comparison)<br>Missing system performance metrics (processing speed, throughput, scalability)<br>Lack of real-world deployment validation beyond controlled user studies	an average of 5 minutes, with a maximum of 80 minutes. For Reddit, the average processing time was 1 minute, with a maximum of 30 minutes. This difference in processing time is attributed to the complexity of the websites; Facebook requires the bot to navigate several additional steps for comment access before data collection, whereas Reddit involves only one to two steps. Furthermore, the speed of Thai word segmentation is slower than that of English.<br><br>To compare the functional differences between our tool and existing ones, we conducted in-depth research, including free tool trials, reviews, and pricing documentation [41, 42, 43, 44]. We observed that most existing tools primarily focus on posts or search terms. While they offer continuous updates, provide demographic data (such as gender, likes, posting time, and hashtags), sentiment analysis, topic analysis, and data insights—functions largely similar to ours—these tools typically require a paid subscription. In addition, this research offers a tool to reduce financial costs, time, and enhance convenience in collecting, analyzing, and presenting information on chronic non-communicable diseases from social media. It also aims to advance education in information science, health information, and data science for broader application.<br>"<br><br>**References Added**<br>41. Talkwalker Case Study. Digital Coach [Internet]. [cited 2025 Jul 14]. Available from: https://www.digital-coach.com/articles/case-studies/talkwalker/<br>42. Pricing. Talkwalker [Internet]. [cited 2025 Jul 14]. Available from: https://www.talkwalker.com/pricing<br>43. Prices. Brand24 [Internet]. [cited 2025 Jul 14]. Available from: https://brand24.com/prices/<br>44. Google Trends. Google [Internet]. [cited 2025 Jul 14]. Available from: https://trends.google.com/trends/
6	Research Contributions Need Clarification: The manuscript still struggles to articulate clear technical innovations	We appreciate the feedback regarding the clarification of our technical innovations. Our work primarily contributes methodological advances by streamlining the entire research workflow. Previously, researchers faced a resource-intensive process involving the manual collection, analysis, and visualization of data. Our tool addresses this by unifying all these methods into a single, integrated platform, thereby offering a distinct

Order	comments	Author reply
	beyond implementation. What specific algorithmic, architectural, or methodological advances does this work contribute to the field?	methodological improvement over fragmented, traditional approaches.<br><br>**Revised manuscript (line 610-617)**<br>"Most current health data analysis still relies on traditional methods such as<br>1.open source platform which not exactly<br>2.this platform utilz AI-powered NLP-enabled LLM which on model<br>3.in this paper we demonstrate overall development & integration of multiple NLP-AI Techniques<br>questionnaires or experiments, which often lead to delays in research and high costs. While utilizing social media data can mitigate these disadvantages, there remains a notable absence of dedicated social media listening tools capable of in-depth health data analysis. Consequently, our tool advances the use of social media data for comprehensive health data analysis, providing results that can be readily summarized or applied to other fields without requiring extensive manual effort from researchers for collection, analysis, and presentation."
7	Limited Scope of Ethical Discussion: While appreciated, the ethics section remains superficial. Given the sensitive nature of health-related social media data, more detailed discussion of data anonymization techniques, consent mechanisms, and privacy-preserving methods is essential.	We have elaborated on our data anonymization techniques and privacy-preserving methods within the 'Data Preprocessing Step' subsection under 'Systems Design'. Regarding consent mechanisms, our approach aligns with GDPR guidelines, which do not necessitate explicit consent for publicly available social media data when deemed not required.<br><br>**Revised manuscript (line 175-178)**<br>"The system cleanses the collected data by removing duplicate comments, filtering out extremely short (less than or equal to 5 words) text entries, and anonymizing personal names by replacing them with Name(n), where 'n' represents the comment number. Our privacy-preserving methods involved removing names from both the database and the web interface, preventing users from extracting and reusing this identifying information. The preprocessed data can be exported in CSV format for future analysis. The tool only scrape data that are shared publicly on the internet"
8	Figure 4 dashboard screenshots remain difficult to read and should be higher	We have optimized the image in this section to achieve the highest possible clarity and have enlarged it to improve ease of viewing and reading. It is recommended that the reader zoom in on the image for enhanced

<table>
<tr><th>Order</th><th>comments</th><th>Author reply</th></tr>
<tr><td></td><td>resolution</td><td>legibility of textual elements.

Origen manuscript
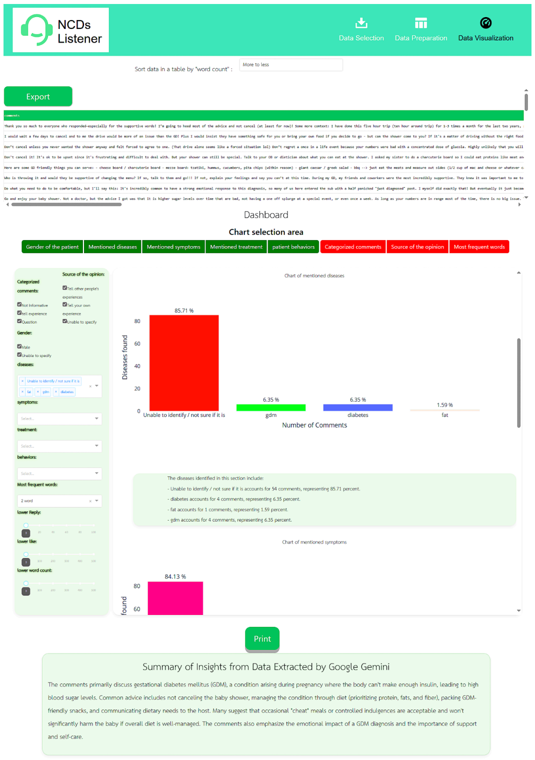

Revised manuscript (Page 17)</td></tr>
</table>

Order	comments	Author reply
		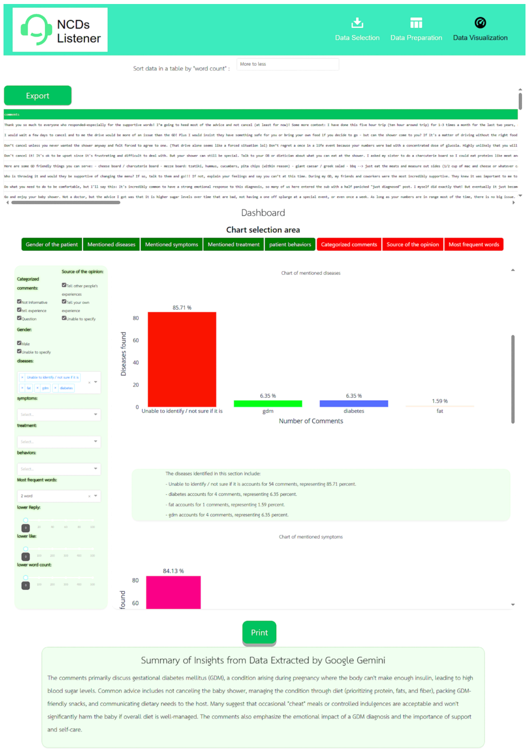
9	The related work section could better position this work within the broader health informatics literature	We have addressed this feedback by expanding the "Related Work" section to better position our work within the broader health informatics literature.<br><br>**Revised manuscript (line 143-155)**<br>"Moreover, this research introduces a tool designed to minimize financial costs, time expenditure, and human resources required for the automated collection, analysis, and presentation of Non-Communicable Disease (NCD) information from social media. This addresses a significant gap, as much existing research in Social Listening for NCDs \cite{b5,b6,b7,b8} typically necessitates considerable resources for data aggregation, analysis, and visualization. While most studies on Applications of Social Media Listening [9-13] have achieved automated data processing and presentation, they often still demand programming expertise for utilization, and some are not directly focused on health content [9,12,13]. This limitation frequently leads to challenges in health data analysis, often yielding only sentiment insights rather than in-depth information such as symptoms or treatment methodologies. Consequently, this tool significantly advances studies in information science, health data science, and related fields,

Order	comments	Author reply
		broadening their potential for wider application.”
10	Some methodological details about web scraping challenges and solutions could be expanded	We have expanded on the methodological details regarding web scraping challenges and solutions in the section titled "Discussion & Conclusion"<br><br>**Revised manuscript (line 586-588)**<br>“Following the first point, since our system relies on URLs from social media platforms that are frequently updated, our web scraping tools must also be continuously adapted. This is particularly critical because social media platforms, especially Facebook, frequently update their HTML tags and implement protective measures against scraping, leading to constant changes in their HTML structure. Therefore, we propose integrating AI to enhance the web scraping process by enabling the AI to autonomously understand the components of the user interface and identify relevant content areas. This approach eliminates the need for constant manual updates to the scraping code.”<br><br>**Revised manuscript (line 623-635)**<br>“Most current health data analysis still depends on traditional methods. Although some open-source platforms do not exactly, they are often not fully suitable for comprehensive health analysis. Certain platforms use AI-powered, NLP-enabled large language models (LLMs), but these typically rely on a single model architecture, which limits their flexibility and depth. In this paper, we demonstrate the development and integration of multiple NLP and AI techniques to overcome these limitations.<br><br>Although social media data has the potential to address some shortcomings of traditional approaches, there remains a significant lack of dedicated social media listening tools capable of conducting in-depth health data analysis. To address this gap, our tool enhances the use of social media data for comprehensive health analysis, providing insights that are easily summarized or applied across various domains without requiring extensive manual effort from researchers for data collection, analysis, and presentation.”

Your sincerely,

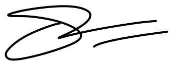

Thanapong Intharah, Ph.D.
Corresponding Author
