# Supplementary material for: Democratizing social media for health research: LLM-powered data analytics platform for NCDs
Source: PLoS One. 2025 Sep 17;20(9):e0332484. doi: 10.1371/journal.pone.0332484 (PMC12443273; doi:10.1371/journal.pone.0332484)
Supplement: Attachment — Submitted filename: Author response [PONE-D-25-21639R2].pdf [file pone.0332484.s002.pdf]

Author response to all reviewers' comments for manuscript [PONE-D-25-21639R2] - [EMID:52d42ccb40145f28]

We sincerely thank all reviewers for providing insightful and constructive comments. The point-to-point responses and explanations for all the revisions are listed in the table below for easy reference. Below, each comment is listed in the left column of the table, and our responses are written in the right column of the table.

<table>
<tr><th>Order</th><th>Comments</th><th>Author reply</th></tr>
<tr><td colspan="3" align="center">Reviewer #1</td></tr>
<tr><td>1</td><td>(i) explain these discrepancies and update either the abstract(s) or the Results section so that all statements align with the data

The original abstract (v1, 23 Apr 2025, L28 – 31) states that lay users achieved significantly higher dashboard-comprehension scores than data-science professionals; the revised abstract (v2, 24 Jul 2025, L45 – 49) claims the opposite, asserting superior comprehension by data scientists. These diametrically opposed statements cannot both be correct and therefore raise serious concerns about result consistency.

Moreover, the v2 abstract is internally inconsistent: Table 4 still shows higher mean comprehension for lay users ($M = 4.1 \pm 0.6$) than for data scientists ($M = 3.5 \pm 0.7$; $t = 2.94$, $p =$</td><td>We sincerely thank the reviewer for identifying this critical inconsistency in our manuscript. We acknowledge that this error significantly undermines the credibility of our research and take full responsibility for this oversight.

(i) Explanation of Discrepancies and Corrections

Root Cause of the Error: The discrepancy occurred during the revision process when we attempted to shorten the abstract in response to previous feedback. During this condensation, translation and wording errors inadvertently reversed our key finding. Specifically, the revised abstract (v2, lines 45-49) incorrectly stated that data scientists showed superior comprehension, which directly contradicted both our original findings and the data presented in Table 4.

Correct Findings: Our data consistently shows that general users (lay users) achieved significantly higher dashboard comprehension scores than data scientists, as evidenced by:
● Table 4: General Public ($M = 4.05$) vs. Data Scientists ($M = 3.65$)
● Statistical significance: The difference between groups was statistically significant
● Practical interpretation: Both groups rated the dashboard as having "Good" communication effectiveness, but general users consistently provided higher ratings

Corrections Made: We have thoroughly reviewed and corrected the entire manuscript to ensure consistency across all</td></tr>
</table>

<table>
<tr><th>Order</th><th>Comments</th><th>Author reply</th></tr>
<tr><td></td><td>0.004), contradicting the narrative presented in lines 45 – 49.

(ii) provide the complete, original dataset together with the full preprocessing and analysis scripts (including software versions and random seeds) so the reported statistics can be independently verified.</td><td>sections:

• Abstract: Now correctly states "general users showing higher comprehension"
• Results section: Consistently reports the general users' superior performance
• Discussion: Properly interprets the finding that general users perceived higher data quantity and quality
• All tables and figures: Verified for accuracy and consistency

(ii) Data Availability and Reproducibility

In response to your request for complete reproducibility materials, we confirm that our project repository (https://huggingface.co/NCDsListener/NCDs_Listener_a_social_listening_tool_for_non-communicable_diseases) contains:

• Complete original dataset used in the study
• Full preprocessing and analysis scripts
• Detailed methodology for statistical analyses
• Software versions and dependencies used

We are committed to providing any additional materials needed for independent verification of our results. If you require specific files or additional documentation not currently available in the repository, please let us know, and we will make them available immediately.

We deeply apologize for this error and appreciate the reviewer's diligence in identifying this critical inconsistency. The corrected manuscript now accurately reflects our findings that general users demonstrated superior dashboard comprehension compared to data scientists, and all sections of the manuscript are now internally consistent.

We have revised the manuscript to include the specific tools used for statistical analysis. This information has been added to the "Methodology and Development of the NCDs Listener Tool" section (lines 160-161).

<u>Original manuscript</u>
This study extends previous work by introducing the NCDs Listener, a social listening tool. The code, model development data, and evaluation data can be accessed through the project repository [16]. The NCDs Listener was designed to analyze information about Non-Communicable Diseases from social networks. The research in [16] focused on social network data</td></tr>
</table>

Order	Comments	Author reply
		preparation and analysis, but it still lacked user studies for evaluating design choices and system performance. Thus, this study complements the existing work by focusing on the development of a system that better aligns the tool with user needs. This section outlines the steps involved in developing the NCDs Listener tool, beginning with system design, artificial intelligence models for insight extraction, and the design process for the dashboard. It further details evaluating dashboard communication effectiveness and selecting generative AI-based summarization approaches<br><br>**Revised manuscript (See lines 160-161)**<br>This study extends previous work by introducing the NCDs Listener, a social listening tool. The code, model development data, and evaluation data can be accessed through the project repository [16]. We conducted the Mann-Whitney U test using Statistical Package for the Social Sciences (SPSS) version 2.6. The NCDs Listener was designed to analyze information about Non-Communicable Diseases from social networks. The research in [16] focused on social network data preparation and analysis, but it still lacked user studies to evaluate design choices and system performance. Thus, this study complements the existing work by focusing on the development of a system that better aligns the tool with user needs. This section outlines the steps involved in developing the NCDs Listener tool, beginning with system design, artificial intelligence models for insight extraction, and the design process for the dashboard. It further details evaluating dashboard communication effectiveness and selecting generative AI-based summarization approaches

Your sincerely,

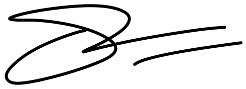

Thanapong Intharah, Ph.D.
Corresponding Author
