# Supplementary material for: Democratizing social media for health research: LLM-powered data analytics platform for NCDs
Source: PLoS One. 2025 Sep 17;20(9):e0332484. doi: 10.1371/journal.pone.0332484 (PMC12443273; doi:10.1371/journal.pone.0332484)
Supplement: Attachment — Submitted filename: Author response[EMID_01a75240c14cced5]-2.pdf [file pone.0332484.s003.pdf]

Author response to all reviewers' comments for manuscript PONE-D-25-21639 - [EMID:0cebbd65333ab58f]

We sincerely thank the reviewers and the editor for the comments. We have carefully considered and addressed all the comments. The point-to-point responses and explanations for all the revisions are listed in the table below.

Order	comments	Author reply
Reviewer #1		
1	Sample representativeness – All 30 usability testers are statistics students (10 were also in the design interviews). Please discuss this limitation explicitly and outline plans to validate the tool with patients and frontline clinicians.	Thank you for your valuable feedback regarding the representativeness of our sample. While we had previously detailed the rationale for our sample selection in the 'Design of the Assessment for Ability and Satisfaction' section (line(s) 412-432) In particular, we explicitly state that the selection of 10 statistics students for the dashboard development phase and 30 statistics students for the evaluation of both the dashboard and the Generative AI was deemed sufficient for initial usability testing. This decision was made to control for variability in baseline knowledge, expedite the development process, and facilitate data collection during the initial phase of the study. We agree that enhancing clarity and transparently addressing the study's limitations is crucial. Therefore, to ensure that readers have a clear understanding of the sample used and the constraints related to its size, we have incorporated the following clarifications into various sections of the manuscript:<br><br>**1. Design of the Assessment for Ability and Satisfaction**<br>**<u>Original manuscript</u>**<br>The study involves a total of 30 volunteers, selected through a combination of stratified sampling and simple random sampling from the list of students in the Department of Statistics, Faculty of Science, Khon Kaen University.<br><br>**<u>Revised manuscript (See lines 412-416)</u>**<br>The study involved a total of 30 volunteers. This cohort was distinct from the 10 volunteers who participated in the initial design interviews for the dashboard. These 30 participants provided an actual power of 81% (as detailed in Table 3). were selected through a combination of stratified sampling and simple random sampling from the

Order	comments	Author reply
		list of students in the Department of Statistics, Faculty of Science, Khon Kaen University.<br><br>**2. Limitation**<br>**<u>Original manuscript</u>**<br>This research focuses on developing an online social media listening tool for Non-Communicable Diseases intended for both the general public and data scientists via web applications. Our goal was to create a tool that meets user needs and provides understandable insights, prioritizing both speed and adherence to methodological principles in its development.<br><br>**<u>Revised manuscript (See lines 600-615</u>**<u>)</u><br>This research focuses on the development of a social media listening tool for Non-Communicable Diseases (NCDs), designed for the general public and data scientists via a web application. The primary goal was to create a tool that meets user needs and provides understandable insights, prioritizing both speed of development and methodological rigor. The user-centered development process involved multiple phases, including an initial dashboard design phase with 10 participants, followed by a testing phase with 30 participants to evaluate dashboard communication effectiveness and select a generative AI-based summarization approach. This study utilized a relatively small dataset from 30 participants in the evaluation phase, all of whom were statistics students from a single institution. While this homogeneous cohort was beneficial for controlled initial usability testing and yielded statistically significant preliminary findings, with a calculated actual power of 81% (as detailed in Table 3), the dataset is too limited in size and diversity to support broad generalizations. The current results should therefore be interpreted strictly as indicative of initial system performance under controlled conditions, rather than as definitive evidence of its effectiveness across diverse populations.<br><br>Regarding the suggestion to outline a validation plan for patients and frontline clinicians, we wish to clarify the scope of the current research. The tool was intentionally developed and evaluated for our primary target audience: the general public and data scientists. We posit that creating a detailed clinical validation plan at this stage would be premature and could detract from ensuring the tool first meets the specific needs of this

Order	comments	Author reply
		initial user base.<br><br>However, validation with clinical populations is a critical next step. As we noted in the Discussion & Conclusion, this is a key component of our future research agenda. To make this planned progression clearer, we have now expanded upon this point in the manuscript as follows:<br><br>**Original manuscript**<br>For future tool development, a re-examination of user needs is crucial, alongside an expansion of evaluation methodologies to include factors such as processing speed, interface aesthetics, functional efficiency, and tool reliability, thereby enhancing the tool's capacity to meet evolving user requirements in the future.<br><br>**Revised manuscript (See lines 715-728)**<br>The present evaluation provides a statistically significant preliminary validation of the tool's usability and communication effectiveness within a controlled academic setting. However, given the limited sample size and the specific background of participants, these results cannot be generalized to the wider population. Future development should therefore be centered on a rigorous re-examination of user needs through expanded evaluation that specifically includes key end-user groups: patients and frontline healthcare professionals. This expanded validation will employ rigorous sampling strategies and a comprehensive evaluation methodology that assesses not only technical performance metrics (e.g., processing speed, functional efficiency) but also user-centric factors such as interface aesthetics, tool reliability, and overall usability. The insights gained from these diverse user groups will be instrumental in developing a strategic implementation roadmap for the tool's adoption in real-world clinical and public health contexts, enabling robust generalization of findings beyond the current controlled academic environment.

2	BERT performance reporting – Report precision, recall, F1, and a confusion matrix on a held-out test set, and describe your train/validation/test split (or cross-validation) strategy.	In response to the reviewer's feedback, we have comprehensively revised the 'Machine Learning for Topic Classification' section to provide greater detail and clarity for the reader. Specifically, we have now incorporated a detailed strategy for our BERT model development, including the data processing pipeline, and have expanded the BERT performance reporting to include a more in-depth analysis.<br><br>**<u>Original manuscript</u>**<br>**Machine Learning for Topic Classification**<br>The system utilizes machine learning models to classify comment types, enabling users to identify comments that are likely to provide valuable information. The BERT model [24] was trained on a dataset of over 3,000 NCD-related comments in Thai and English,<br><br>For model training, we used a dataset of comments (text) extensively collected from social media and categorized comments into non-informative, sharing experience, and inquiring. The consensus among the three annotators was used as the label. The dataset was then split into 70% for training and 30% for testing. To address the class imbalance in the training data, we applied oversampling using the imbalanced-learn Python package. Data preparation involved Tokenization and Stop Words removal. For this training, we employed transfer learning from pre-trained models, utilizing wangchanberta-base-att-spm-uncased for the Thai language [25] and bert-large-uncased for the English language [26]. We trained a BERT model with a maximum sequence length of 200 characters and a learning rate of 2×10-5. Early Stopping was employed to mitigate overfitting during the training process effectively [27,28]. Separate Thai and English models were developed using these identical parameters. Both the Thai and English models demonstrate over 80\% accuracy. This strong performance enables the model to classify comments into three distinct categories effectively:<br><br>Non-informative: Comments that are not related to NCDs or lack meaningful information, such as simply stating the name of a disease or unclear descriptions of symptoms<br><br>Sharing experience: Comments that share personal experiences with diseases, including symptoms (before or after diagnosis), treatments, and how the disease has

Order	comments	Author reply
		affected the quality of life.<br><br>Inquiring: Comments that inquire about various aspects of NCDs, such as symptoms, treatments, or disease impacts.<br><br>Of course. Here is the text with all LaTeX formatting and highlighting removed, converted into plain text suitable for general use.<br><br>**Revised manuscript (See lines 217-317)**<br>**Machine Learning for Topic Classification**<br>This section details the methodology for the development and evaluation of the BERT model [24] for topic classification. It outlines the complete model development pipeline and presents a comprehensive performance analysis, including an interpretation of the confusion matrix (presented in Figure 2) to assess classification accuracy.<br><br>**BERT Model Output Categories**<br><br>The fine-tuned BERT model serves as an automated classifier, designed to categorize social media comments with high efficacy. This classification enables a structured analysis of public discourse surrounding NCDs. The model effectively assigns each comment to one of the following three predefined categories:<br><br>Non-informative: Comments that are not related to NCDs or lack meaningful information, such as simply stating the name of a disease or unclear descriptions of symptoms. This category is used to filter out such data, thereby improving the accuracy and relevance of the final results.<br><br>Sharing experience: Comments that share personal experiences with diseases, including symptoms (before or after diagnosis), treatments, and how the disease has affected the quality of life.<br><br>Inquiring: Comments that inquire about various aspects of NCDs, such as symptoms, treatments, or disease impacts.<br><br>**The model development process was conducted in 4 sequential stages:**<br><br>1. **Step 1: Dataset Collection and Annotation**

Order	comments	Author reply
		A dataset comprising over 3,000 comments related to Non-Communicable Diseases (NCDs) was extensively collected from social media platforms. This dataset was then manually categorized into three distinct classes: “non-informative”, “sharing experience”, and “inquiring”. To ensure high-quality and reliable ground-truth data, the final label for each comment was determined by achieving a consensus among three expert annotators.<br><br>2. **Step 2: Data Partitioning and Preprocessing**<br>For robust model evaluation, the annotated dataset was partitioned into a 70% training set and a 30% held-out test set using the train_test_split function from the scikit-learn library in Python. This division ensures that the model’s final performance is assessed on unseen data, providing an unbiased measure of its generalization capability. To mitigate the effects of class imbalance within the training data, an oversampling technique was applied using the “imbalanced-learn” Python package. Subsequently, the raw text underwent a data preparation pipeline involving two key steps: tokenization and stop-word removal. These procedures were performed using the PyThaiNLP library for Thai text and the Natural Language Toolkit (NLTK) for English text.<br><br>3. **Step 3: Model Selection via Transfer Learning**<br>We employed a transfer learning approach to leverage the extensive linguistic knowledge encapsulated in large pre-trained language models. For the Thai language classifier, the “wangchanberta-base-att-spm-uncased” model was selected as the foundational architecture [25]. For the English language classifier, the “bert-large-uncased” model was utilized [26].<br><br>4. **Step 4: Model Fine-Tuning and Training**<br>Separate models for the Thai and English languages were fine-tuned using identical hyperparameters. Each BERT-based model was trained with a maximum sequence length of 200 characters and a learning rate of 2 x 10^-5. To prevent overfitting and ensure the model generalized well, an *Early Stopping* mechanism was implemented. This technique effectively concludes the training process when performance on a validation set ceases to improve [27, 28]. The performance of the resulting fine-tuned models was then comprehensively evaluated on the 30% held-out test set using Python's scikit-learn library to calculate key performance metrics, including accuracy, precision, recall, and F1-score. These

Order	comments	Author reply
		evaluation results are detailed in Tables 1 and 2.<br><br>Thai Model Performance<br><br>As shown in the results table, the Thai BERT model demonstrated exceptionally high performance for two of the three categories.<br><br>The "Sharing experience" class achieved an F1-score of 0.95, with relatively strong Precision (0.97) and strong Recall (0.94), suggesting that the model captures most instances of this class while maintaining reasonable accuracy. The "Non-informative" class performed the best overall, with an F1-score of 0.98, benefiting from near-perfect Precision (0.98) and high Recall (0.99).<br><br>Conversely, the "Inquiring" class proved to be the most significant challenge, with a low F1-score of 0.40. This score reveals a substantial weakness in the model's ability to handle questions.<br><br>Low Recall (0.40): This is the most critical issue. It signifies that the model correctly identified only 40% of all actual "Inquiring" comments in the test set. The confusion matrix would show that the remaining 60% (False Negatives) were predominantly misclassified as "Sharing experience" or "Non-informative". This suggests a significant linguistic ambiguity where questions in Thai social media are often embedded within narrative text, making them difficult for the model to distinguish.<br><br>Low Precision (0.40): This indicates that when the model predicted a comment was "Inquiring", it was correct only 40% of the time. This is due to a high number of False Positives, where comments from the other two classes were incorrectly labeled as "Inquiring".<br><br>English Model Performance<br><br>The English BERT model exhibited a similar performance pattern, though the challenge with the "Inquiring" class was less severe (see results table).<br><br>The model performed well on the "Sharing experience" and "Non-informative" classes, achieving F1-scores of 0.78 and 0.85, respectively. This indicates robust and reliable classification performance for the majority of comments, with particularly strong Precision on the

Order	comments	Author reply
		"Non-informative" class (0.88).<br><br>The "Inquiring" class remained the weakest, with an F1-score of 0.56.<br><br>Moderate Recall (0.60): The model successfully identified 60% of all true "Inquiring" comments, a considerable improvement over the Thai model. However, the confusion matrix would still show a notable number of False Negatives, where nearly 40% of questions were missed.<br><br>Moderate Precision (0.53): With a Precision of 0.53, about half of the comments that the model labeled as "Inquiring" were indeed questions. The other half were False Positives, indicating that the model still struggles with comments that have question-like features but are not explicit inquiries. The primary source of confusion was with the "Sharing experience" class, where comments with question-like features were often misclassified.<br><br>In summary, the fine-tuned BERT model demonstrated strong overall performance across both test sets, achieving high accuracy in classifying “Sharing experience” and “Non-informative” categories, with consistently elevated precision, recall, and F1-scores. However, performance was notably lower for the “Inquiring” category, with comparatively reduced precision, recall, and F1-scores, indicating that this class remains more challenging for the model to predict accurately. These results suggest that while the model is effective in identifying the majority of content types, further refinement is required to improve its ability to recognize inquiring statements.<br><br>To provide a more detailed analysis of model performance, we have included **confusion matrix tables** and **performance evaluation tables** for both the Thai and English models in the manuscript.

Order	comments	Author reply

Revised manuscript (See page 8)

Confusion matrix tables (See Figure 2)

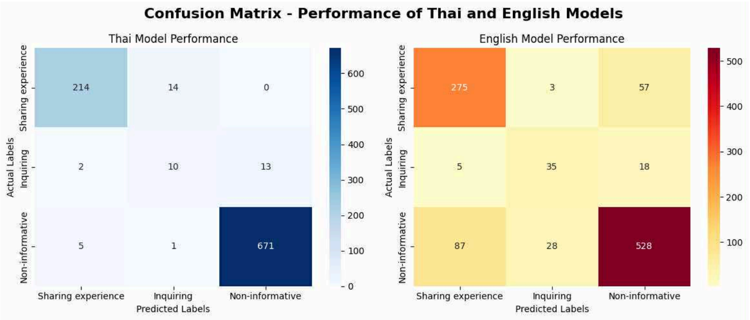

Confusion matrices of classification performance for the Thai and English models. The matrices illustrate the performance on the hold-out test sets for the Thai model (left), evaluated on 930 instances (30% of the Thai test data), and the English model (right), evaluated on 1,036 instances. The diagonal cells represent the number of correctly classified instances for each class, while off-diagonal cells indicate misclassifications. Both models demonstrate strong predictive accuracy, particularly for the "Sharing experience" and "Non-informative" classes. A comprehensive breakdown of performance metrics, including precision, recall, and F1-scores, is provided in Table 1 (Thai) and 2 (English).

Performance evaluation tables (See Table 1 and Table 2)

Table 1. Performance Metrics for the Fine-Tuned BERT Model on the Thai Test Set. Overall Accuracy: 96.24%.

Category	Precision	Recall	F1-score
Sharing experience	0.97	0.94	0.95
Inquiring	0.40	0.40	0.40
Non-informative	0.98	0.99	0.98

Table 2. Performance Metrics for the Fine-Tuned BERT Model on the English Test Set. Overall Accuracy: 80.89%.

Category	Precision	Recall	F1-score
Sharing experience	0.75	0.82	0.78
Inquiring	0.53	0.60	0.56
Non-informative	0.88	0.82	0.85

Based on the present findings, we have extended our work toward enhancing the granularity of the model's

Order	comments	Author reply
		output and advancing the development of content-type classification models to achieve greater efficiency in future research.<br><br>● Enhancing the granularity of the model's output<br><br>**Original manuscript**<br>The BERT model employed in this study has been trained on over 3,000 datasets and demonstrates an accuracy of 80% in processing both Thai and English data.<br><br>**Revised manuscript (See lines 636-638)**<br>The BERT model employed in this study has been trained on over 3,000 datasets and demonstrates high accuracy, exceeding 80% for both Thai and English data; however, it still presents limitations in classifying text in the "Inquiring" category.<br><br>● Future research<br>**Revised manuscript (See lines 729-736)**<br>In terms of artificial intelligence models for insight extraction, several key areas warrant further investigation. For machine learning-based topic classification, future research should focus on enhancing the model's ability to distinguish between different question types through architectural improvements and expansion of the test dataset to achieve greater statistical power. Critically, bias reduction in data classification represents a priority area, necessitating a transition from human-annotated opinion characteristics to automated machine learning or deep learning approaches for feature identification and classification.
3	LLM-generated summary evaluation – Complement participant Likert ratings with an objective metric (e.g., ROUGE/BLEU or medical-entity coverage) or clinician expert review with inter-rater agreement.	We thank the reviewer for their insightful comments. We would like to clarify the context for our current design choices. Our primary target audience at this stage of development consists of the general public and data scientists. Consequently, the principal objective of the LLM-generated summary is to render complex social media data on non-communicable diseases (NCDs) accessible to users who may lack expertise in graph theory or data analysis.<br>Regarding the valid concern about the tool's reliability, we acknowledge that the output's fidelity is inherently dependent on the quality of the user-provided input data—a well-documented challenge in social media analytics [1]. Currently, there are no established gold

Order	comments	Author reply
		standards for evaluating the quality and accuracy of social media-derived NCD data summaries, which presents a fundamental challenge in validation. To mitigate reliability concerns within this limitation, our methodology incorporates a two-pronged approach:<br><br>1. A BERT model is employed to pre-process and filter out non-informative or low-utility data.<br><br>2. A Retrieval-Augmented Generation (RAG) framework is utilized to ensure the LLM grounds its summaries strictly on the provided, filtered data, thereby preventing confabulation or the generation of unsupported information.<br><br>Nevertheless, we appreciate this feedback and, as noted in the manuscript, we will incorporate these considerations into the future development of our tool. Future work will prioritize the development of domain-specific gold standards and validation methodologies tailored to social media NCD data analysis, which will enable more rigorous evaluation of our tool's performance and reliability.<br><br>To enhance the clarity and transparency of our manuscript for the reader, we have made the following key revisions:<br><br>**1. We have specified the tools and methodologies used for identifying and filtering irrelevant data.**<br>**Original manuscript**<br>Non-informative: Comments that are not related to NCDs or lack meaningful information, such as simply stating the name of a disease or unclear descriptions of symptoms<br><br>**Revised manuscript (See lines 228-229)**<br>Non-informative: Comments that are not related to NCDs or lack meaningful information, such as simply stating the name of a disease or unclear descriptions of symptoms. This category is used to filter out such data, thereby improving the accuracy and relevance of the final results.<br><br>And<br><br>**Original manuscript**<br><br>This RAG approach is applied to ensure domain-specific accuracy and mitigate hallucinations within the generative

Order	comments	Author reply
		AI component of the NCDs Listener system.<br><br>**<u>Revised manuscript (See lines 326-327)</u>**<br>This RAG approach is applied to ensure domain-specific accuracy and to prevent the model from generating fabricated information (i.e., hallucinations) within the generative AI component of the NCDs Listener system.<br><br>**2. We have explicitly stated the primary objective for implementing the Large Language Model (LLM).**<br><br>**<u>Original manuscript</u>**<br>We integrate generative AI for comment summarization, employing the Google Gemini 2.0 Flash model through LangChain [29]. We utilize LangChain as our Generative AI framework, which facilitates calls to the Google Gemini API and structures summarization as desired by the user. The Google Gemini 2.0 Flash model serves as the model for summarizing sentiment, employing the Retrieval-Augmented Generation (RAG) approach by leveraging data from a vector database. These vectors are generated by embedding all user-desired comment texts. This RAG approach is applied to ensure domain-specific accuracy and mitigate hallucinations within the generative AI component of the NCDs Listener system. The question-answering capability of generative AI is based on a predefined set of questions to guide Gemini in generating summaries.<br><br>**<u>Revised manuscript (See lines 329-332)</u>**<br>We integrate generative AI for comment summarization, employing the Google Gemini 2.0 Flash model through LangChain [29]. LangChain serves as our Generative AI framework, facilitating calls to the Google Gemini API and structuring summarization according to user specifications. The Google Gemini 2.0 Flash model is utilized for sentiment summarization, employing a Retrieval-Augmented Generation (RAG) approach by leveraging data from a vector database. These vectors are generated by embedding all user-desired comment texts. This RAG approach is applied to ensure domain-specific accuracy and prevent the model from generating fabricated information (i.e., hallucinations) within the generative AI component of the NCDs Listener system. The generative AI's question-answering capability is based on a predefined set of questions to guide Gemini in generating summaries. **The primary objective of these LLM-generated summaries is to**

Order	comments	Author reply
		**enable users without expertise in graph analysis or data analytics to comprehend content related to non-communicable diseases (NCDs) within online social media.**"<br><br>**3. We have incorporated a discussion on the inherent limitations of using social media data.**<br>**Original manuscript**<br>Currently, our tool supports content in both Thai and English, and it is designed to extract public comments exclusively from two social media platforms: Reddit and Facebook. The tool demonstrates optimal effectiveness when applied to comment data related to Non-Communicable Diseases (NCDs). Furthermore, as our web application processes comments and chat-like messages, it encounters inherent variations in language, writing style, and terminology. While this approach allowed us to develop the tool effectively within our limitations, it is important to note that these factors may lead to some variations in the accuracy of the results.<br><br>**Revised manuscript (See lines 621-622, 624-626)**<br>Currently, our tool supports content in both Thai and English and is designed to extract public comments exclusively from two social media platforms: Reddit and Facebook. The tool demonstrates optimal effectiveness when applied to comment data related to Non-Communicable Diseases (NCDs). Furthermore, as our web application processes comments and chat-like messages, it encounters inherent variations in language, writing style, and terminology. **The nature of discussions within a post also influences the results.** While this approach allowed us to develop the tool effectively within our limitations, it is important to note that these factors may lead to some variations in the accuracy of the results. **Additionally, the input of unreliable data by users can impact the results, even with the assistance of our BERT model, designed to filter uninformative content.**<br><br>**4. We have expanded the 'Future Work' section to outline our plans for enhancing the tool's medical credibility.**<br>**Revised manuscript (See lines 715-728)**<br>The present evaluation provides a statistically significant preliminary validation of the tool's usability and communication effectiveness within a controlled academic setting. However, given the limited sample size

Order	comments	Author reply
		and the specific background of participants, these results cannot be generalized to the wider population. Future development should therefore be centered on a rigorous re-examination of user needs through expanded evaluation that specifically includes key end-user groups: patients and frontline healthcare professionals. This expanded validation will employ rigorous sampling strategies and a comprehensive evaluation methodology that assesses not only technical performance metrics (e.g., processing speed, functional efficiency) but also user-centric factors such as interface aesthetics, tool reliability, and overall usability. The insights gained from these diverse user groups will be instrumental in developing a strategic implementation roadmap for the tool's adoption in real-world clinical and public health contexts, enabling robust generalization of findings beyond the current controlled academic environment.<br><br>5. Given the importance of employing additional evaluation metrics to further enhance the performance and accuracy of LLMs, we propose that future research will focus on this aspect once the target population has been expanded to include both healthcare professionals and patients.<br><br>**Revised manuscript (See lines 737-752)**<br>Regarding large language model development, the incorporation of additional performance metrics beyond current measures is essential for comprehensive evaluation. While standard text generation metrics such as ROUGE and BLEU scores are widely used in natural language processing, there are currently no established gold standards specifically designed for evaluating social media-derived non-communicable disease (NCD) data summaries. This limitation extends even to the application of conventional metrics like ROUGE and BLEU scores, as appropriate reference standards for our specific domain remain undefined.<br>Future implementations should therefore focus on developing domain-specific evaluation frameworks that incorporate adapted versions of ROUGE and BLEU scores alongside medical-entity coverage metrics, particularly as the target population expands to include both patients and healthcare providers. A critical priority for future work will be establishing gold standards tailored to social media NCD analytics, which will enable the creation of robust evaluation methodologies specific to our application domain. These enhancements will enable

Order	comments	Author reply
		more comprehensive and contextually appropriate evaluation frameworks, supporting the model's applicability and validation across diverse clinical contexts.<br><br>1.Kshavarz, H. (2021). Evaluating credibility of social media information: current challenges, research directions, and practical criteria. *Information Discovery and Delivery*, *49*(4), 269–279. https://doi.org/10.1108/IDD-03-2020-0033
4	Data & code availability – PLOS ONE requires open data at submission. Please deposit all anonymized raw data, analysis scripts, and dashboard configuration files in a public repository and cite the DOI.	We want to reiterate and confirm that the data used in our research is publicly accessible at [Harvard Dataverse>>> NCDs Listener: A Social Listening Tool for Non-Communicable Diseases] and the dataset at URL: [https://doi.org/10.7910/DVN/HRSM09]. The repository contains: (1) anonymized raw data used for model creation, (2) query data for graph and variable analysis, and (3) query data supporting the communication functions of both the Dashboard and the Generative AI. All datasets have been thoroughly anonymized before publication. The data availability statement is added to the manuscript.
5	Figure 2: Visual quality – The current visualization is too minimal; enhance labels, legend, and contextual information so readers can interpret the results at a glance.	Thank you for your valuable suggestion. We have enhanced the resolution of Figure 3<br><br>**<u>Original manuscript</u>**<br>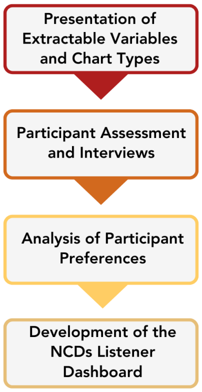<br><br>**<u>Revised manuscript (See Figure 3, page 12)</u>**

Order	comments	Author reply
		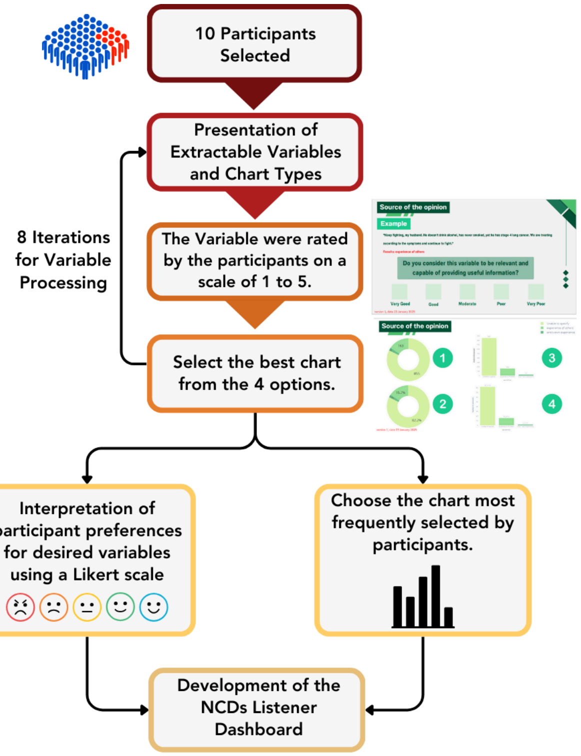<br><br>We have also expanded on the *'Dashboard Design Process'* to include a more detailed description of the participants from the data scientist group and their specific role in selecting charts and graphs, along with enhancing the figure captions to convey the maximum level of detail and clarity.<br><br>• Detailed description of the participants<br>**Original manuscript**<br>5 data scientists or students from the same department who had completed the course<br><br>**Revised manuscript (See line 359-360)**<br>5 data scientists or students from the same department who had completed the course and had shown a high level of proficiency in the subject matter<br><br>• Figure captions to convey the maximum level of detail and clarity<br>**Original manuscript**<br>**The dashboard design process** commenced with the presentation of knowledge extraction variables and the designed chart types. Subsequently, participants were assessed and interviewed to analyze their needs, and the collected data were utilized to develop the NCDs Listener

Order	comments	Author reply
		dashboard.<br>**<u>Revised manuscript (See Figure 3, Page 12)</u>**<br>**The dashboard design process.** The process began with the selection of 10 participants. Each was presented with a set of extractable variables and four chart types for visualization. For every variable, participants rated its importance on a 1–5 Likert scale and selected the best chart from the four options presented. This was repeated for eight variables. Ratings were analyzed to interpret preferences for desired variables, while chart type preferences were determined by identifying the most frequently selected option. The results of these evaluations were used to guide the presentation and design of the final visualizations. These visualizations were then integrated into the development of the NCDs Listener dashboard, ensuring that both content and presentation reflected participant preferences.<br><br>To facilitate readers' ability to quickly and clearly understand the details presented in each figure, we have incorporated distinctive markers and expanded the figure captions to offer the maximum possible level of detail.<br><br>● **<u>Figure 1</u>**<br>**<u>Change Figure</u>**<br>**<u>Original manuscript</u>**<br>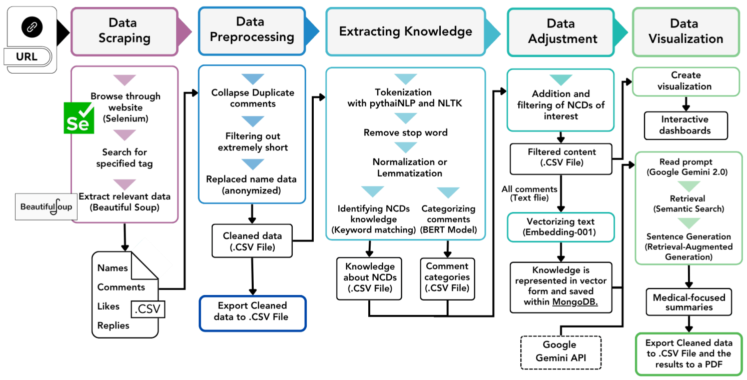<br>**<u>Revised manuscript (See Figure 1, Page 6)</u>**

Order	comments	Author reply
		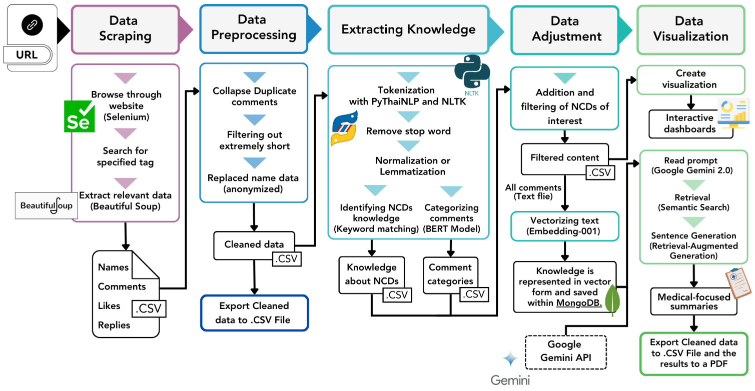<br><br>**<u>Change captions</u>**<br>**<u>Original manuscript</u>**<br>**Systems design** The process begins with data collection from social media, followed by data cleaning and knowledge extraction. Users can then refine the data according to their specific needs, with the final results presented through a dashboard and summarized using generative AI.<br><br>**<u>Revised manuscript (See Figure 1, Page 6)</u>**<br>**System design and workflow for social media–based NCD monitoring and summarization.** The workflow consists of five stages: (1) Data Scraping – Social media data are collected using Selenium and BeautifulSoup, extracting relevant elements (names, comments, likes, replies) into CSV files. (2) Data Preprocessing – Duplicate and extremely short comments are removed, and personal identifiers are anonymized. (3) Extracting Knowledge – Cleaned text is tokenized, stop words removed, and normalized or lemmatized; NCD-related content is identified via keyword matching, and comment categories are classified using a BERT model. (4) Data Adjustment – Content is refined for NCDs of interest, vectorized (Embedding-001), stored in MongoDB, and processed with the Google Gemini API. (5) Data Visualization – Interactive dashboards present results, with retrieval-augmented generation producing summaries and medical-focused narratives. Final datasets and reports are exportable in CSV and PDF formats.

Order	comments	Author reply
		• **Figure 4**<br>**Change Figure**<br>**Original manuscript**<br>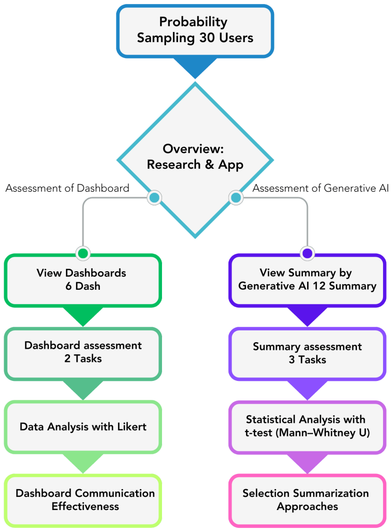<br><br>**Revised manuscript (See Figure 4, Page 15)**<br>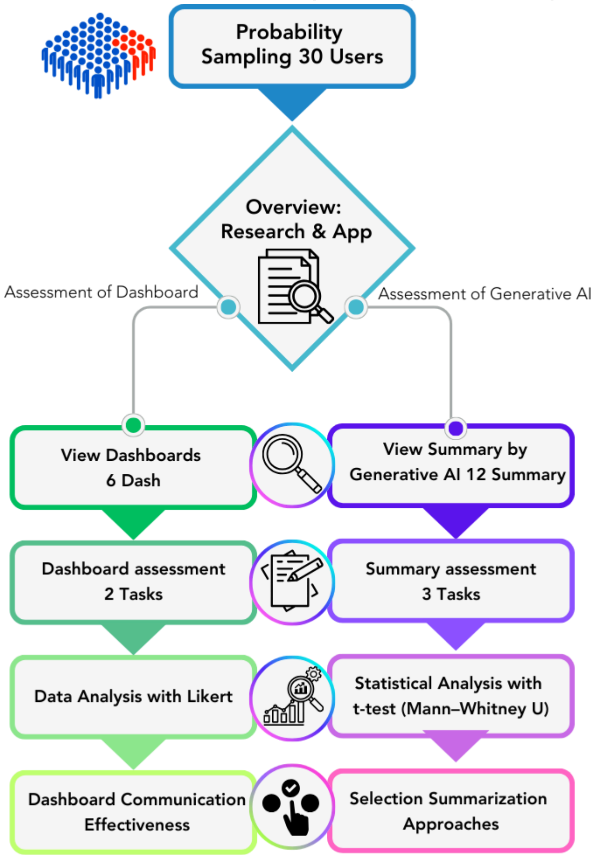

Order	comments	Author reply
		**Change captions**<br>**Original manuscript**<br>**Evaluation strategy** entails collecting data from user groups to assess the dashboard's communicative effectiveness and to determine an appropriate approach for summarizing generative AI content. The analysis will utilize Likert scale measurements and a Mann–Whitney U for statistical evaluation.<br><br>**Revised manuscript (See Figure 4, Page 15)**<br>**Evaluation strategy** illustrates the process for assessing both the communicative effectiveness of the dashboard and the optimal summarization approach for generative AI content. A probability sample of 30 users was selected and divided into two main assessment paths. The first path involved evaluating six dashboards through two assigned tasks, followed by data analysis using a Likert scale to measure user perceptions, ultimately determining the dashboard's communication effectiveness. The second path focused on assessing generative AI summaries, where participants reviewed twelve AI-generated summaries and completed three evaluation tasks. Statistical comparisons were conducted using the t-test and Mann–Whitney U test to identify significant differences in performance and preferences. The combined results from both assessments informed the selection of the most suitable summarization approaches and guided improvements in dashboard presentation and AI integration.<br>• **Figure 5**<br>**Change captions**<br>**Original manuscript**<br>**The NCDs Listener dashboard results** include an editable and exportable raw data table at the top, a dashboard customization area on the left, a chart and legend display area on the right, and a generative AI-generated summary of overall content at the bottom.<br><br>**Revised manuscript (See Figure 5, Page 20)**<br>**NCDs Listener Example dashboard interface displaying comprehensive analytical results and data visualization capabilities.** The dashboard features an editable and exportable

Order	comments	Author reply
		raw data table at the top, enabling direct data manipulation and export capabilities. The left panel presents a customizable dashboard area with interactive toggle switches displayed in green (active/enabled) and red (inactive/disabled) states for dynamic parameter control. The central visualization area displays statistical charts and graphical representations of the processed NCD data. The bottom of the chart contains a legend and a data display area for a better understanding of the data. At the bottom, an AI-generated summary section provides automated insights from Google Gemini integration, offering intelligent analysis and interpretation of the uploaded content. This integrated interface facilitates comprehensive non-communicable disease data analysis through intuitive visualization, real-time parameter adjustment, and automated analytical summaries for enhanced research workflow efficiency.
